# Comparative Mortality

**Published:** 1909-11-20

**Authors:** 


### Comparative Mortality.

In a paper read before a recent meeting of the Scottish Faculty of Actuaries, Dr. Dunlop brings forward some striking figures to illustrate his views on occupation mortalities. Taking the large mass of statistical material that has accumulated in the Registrar-General's Office as a basis for his deductions, he has collated with it the results of former tabulations made in America, France, and other countries. The method he employed to arrive at his own conclusions is laborious and scientifically more or less accurate, but it is one that appeals more to an expert statistician, such as one imagines a member of the Faculty before which he read his paper to be, than to the average medical man, whose experience of statistics, as Dr. Hale White pointed out two years ago, is usually of the slightest. Taking the expectations of life at the age of 25, Dr. Dunlop finds that clergymen stand first on the list with 42.8 years—a conclusion which is consonant with the results of other investigators. Then come gardeners (42.3), gamekeepers (42.1), farmers, engine-drivers, farm labourers, schoolmasters, and brickmakers—all open-air livers with one class exception—whose expectation exceeds 41 years. The twelve occupations that show the highest mortality, and therefore the lowest expectation (27 to 33 years), are, in ascending order, general labourers, tin miners, costermongers, hotel servants, publicans, seamen, filemakers, shopkeepers, cutlers, dock labourers, messengers, and potters. From these tables it is evident that "unskilled labour" occupations have a higher mortality figure than those of which the members are more selected, physically and mentally, though the rule does not apply in every case. Open-air trades, again, compare very favourably with occupations in which the workers have to labour in confined space, to breathe impure air, and to live lives of uncertainty and worry.

The constant rush and anxiety that the average doctor has to endure, for example, are responsible no doubt for the high mortality figure that has been ascribed to the medical profession, notwithstanding the fact that practitioners, as a class, are not careless of hygienic rules even though they may have a predilection for keeping their house windows shut. In discussing these figures it should be remembered that Dr. Dunlop's basis—25 years—is fairly low. There is no profession or occupation that entails so long and arduous an apprenticeship as that of medicine, and no apprenticeship that subjects the beginner to so much risk of losing health and strength. The clergyman, no matter to what sect or denomination he belongs, has a comparatively smooth and uneventful student life; his risks only commence after he has entered into his own, when his occupation entails the breathing of vitiated church or slum atmospheres. The practitioner, on the other hand, spends the greater part of his student life in the wards or the still more impure air of the out-patient department; he is constantly and continually exposed to infection, and few students go through their course without becoming victims temporarily to their environment. Finally, when qualified and in practice, the doctor has a life of even greater stress and strain before him, and it is not to be wondered at that his expectations are scarcely much higher than those of the manual labourer, whose high mortality figure is accounted for by precisely the same factors—excessive strain, irregularity, and hard work.

